# Psychosomatic symptom burden and family functioning of adolescents with chronic diseases in transition to adult medicine – Implications for psychosocial support

**DOI:** 10.1371/journal.pone.0359441

**Published:** 2026-09-30

**Authors:** Daniela Choukair, Amelie Beuchert, Cinara Paul, Alexander Fichtner, Georg F. Hoffmann, Hans-Christoph Friederich, Beate Wild

**Affiliations:** 1 Department of Pediatrics I, Medical Faculty Heidelberg, Heidelberg University, Heidelberg, Germany; 2 Center for Rare Diseases, University Hospital Heidelberg, Heidelberg, Germany; 3 Department of General Internal Medicine and Psychosomatics, University Hospital Heidelberg, Heidelberg, Germany; 4 Institute for General Practice and Interprofessional Care, Tübingen University Hospital, Tübingen Germany; Medical University of Vienna, AUSTRIA

## Abstract

**Purpose:**

Adolescents with chronic diseases often experience psychosomatic comorbidity. However, systematic evaluation of psychosocial stress is not yet implemented in structured transition programs from pediatric to adult health care. The aim was to evaluate psychosocial burden, family functioning, knowledge of their illnesses and identify needs for support.

**Methods:**

Among n = 92 adolescents with chronic diseases psychosocial stress and family functioning were assessed using validated questionnaires at the beginning of transition: (Patient Health Questionnaire (PHQ-9), Generalized Anxiety Disorder Scale-7 (GAD-7), 8-item Somatic Symptom Scale (SSS-8), Somatic Symptom Disorder-B Criteria Scale (SSD-12), Systemic Clinical Outcome and Routine Evaluation (SCORE-15)). The patient´s disease-specific knowledge and need for counselling were assessed using a transition questionnaire. Semi-structured interviews were conducted in 42 adolescents with increased psychosocial burden and analysed using qualitative content analysis by Kuckartz.

**Results:**

39.6% (95% CI = 29%, 50%) were moderately impaired by somatic complaints (SSS-8) and 6.5% (95% CI = 1%, 12%) raised severe concerns due to somatic symptoms (SSD-12). 20.6% (95% CI = 10.3%, 28%) showed symptoms of moderate or severe depression (PHQ-9), whereas 11.9% (95% CI = 6.8%, 17.5%) suffered from moderate or severe generalized anxiety disorder symptoms (GAD-7). Family functioning was assessed as good (SCORE-15); and a lower family functioning was significantly associated with higher psychosomatic symptom severity (p < 0.05). Qualitative analysis identified four main categories: somatic complaints, psychological aspects, informational aspects, and structural problems.

**Discussion:**

At the beginning of the transition, adolescent patients with chronic diseases suffered from psychosomatic burden and a lower family functioning was associated with higher psychosomatic symptom severity. Therefore, assessment of psychosocial burden and family functioning should be integrated into transition programs to provide specific support and initiate early interventions.

## Introduction

The physical, social, and cognitive changes in adolescence lead to a completely new appraisal of one´s environment and the importance of various issues [1]. This time period in life is particularly challenging for adolescents and young adults with chronic illnesses, which can result in discontinuation of treatment, inadequate care, or complications from the underlying illness. According to a national survey in Germany about 15% of people under 18 are affected by a chronic disease that requires special care [2]. They have to cope with disease-specific morbidity and sequelae such as altered body composition, chronic pain, and potential limitations in their quality of life [3]. This can cause fear and uncertainty. It is important for children to have caregivers who are supportive, engaged, and responsive to build resilience and secure attachment. However, the bio-psycho-social situation of a family with a chronically-ill child can be extremely burdened and stressful, depending on the severity and prognosis of the disease as well as on the coping resources of the family [4].

Over the last decades, survival of children and adolescents with chronic diseases has considerably increased. Consequently, many of these patients move continuously from pediatric into adult health care services [5,6]. To date, it has been shown that a structured and well supported transition process is imperative for further development, treatment, and outcome [7].

However, the transition process also depends on the affected adolescents and their families. Psychosocial burden and family functioning are thought to be important factors facilitating a successful transition process [8]. It has been shown that 20–50% of adolescents with a chronic condition experience psychological comorbidity, which is twice as high as in healthy young people [9]. However, to date, systematic evaluation of psychosocial stress is not yet implemented in transition pathways. Consequently, appropriate support services are often not provided.

This is the first study to investigate the psychosocial burden and family functioning in adolescents with chronic diseases at the beginning of the transition period. Validated questionnaires and a semi-structured interview were used to learn more about the young people’s bio-psycho-social situation and to provide specific support and initiate early interventions.

## Methods

### Study design

The data collection was conducted using a mixed-methods approach. A questionnaire was used to collect quantitative data, including validated questionnaires assessing psychosocial stress, family functioning and sociodemographic data. Participants were recruited between 24^th^ August 2022 and 12^th^ March 2023 and included adolescents aged ≥ 16 years with chronic diseases in the fields of gastroenterology, nephrology, endocrinology, pulmonology, and rheumatology. Participants were invited to take part in the study and complete the questionnaire during an outpatient appointment or a hospital stay. Inclusion criteria were at least 16 years of age, the presence of a chronic disease, and written informed consent (of a parent or guardian if the participant was under 18 years old). During this period, 114 adolescents were invited, 103 of whom agreed to participate. Five adolescents were unable to complete the questionnaire independently due to cognitive impairment. A total of 98 questionnaires were completed, however six had to be excluded due to a lack of parental consent. Therefore, 92 questionnaires were included in the analysis. Forty-nine adolescents completed an additional transition questionnaire assessing the disease-specific knowledge and counseling needs [7].

### Research questions

The aim of this study was to evaluate psychosocial burden, family functioning, and knowledge of their specific illnesses in chronically ill adolescents and identify support needs at the beginning of the transition period.

### Data collection tools

#### Questionnaires.

Depression was assessed by using the nine-item depression module of the Patient Health Questionnaire (PHQ-9) [10]. The PHQ-9 contains nine items that correspond to the diagnostic DSM-V criteria for a depressive disorder. PHQ-9 scores range from 0 to 27. Scores of 5–9, 10–14, and ≥ 15 represent mild, moderate, and severe depression, respectively [10]. Test-retest reliability of the PHQ depression module ranges between 0.81 and 0.96 [11]. A cut-off of ≥11 has demonstrated a sensitivity of 98% and specificity of 80% to detect a depressive episode [11].

The seven-item Generalized Anxiety Disorder Scale (GAD-7) was used to measure symptoms of generalized anxiety disorder over the past two weeks [12]. GAD-7 scores range from 0 to 21. Scores of ≥ 5, ≥ 10, and ≥ 15 represent mild, moderate, and severe anxiety symptom levels, respectively. A score of ≥ 10 is considered the cut-off point for generalized anxiety disorder [12]. Test-retest reliability of the GAD-7 was estimated to be 0.83 [12].

The eight-item Somatic Symptom Scale (SSS-8) was used to measure somatic symptom burden over the past seven days [13,14] and included assessment of pain, fatigue, gastrointestinal, and cardiopulmonary symptoms as part of the general somatic symptom burden. Scores range from 0 to 32, with higher scores indicating a greater symptom burden. A cut-off ≥ 8 indicates at least moderate somatic symptom burden [13]. The SSS-8 is a widely used instrument with a good reliability (Cronbach α = .81) [13].

The Somatic Symptom Disorder-B Criteria Scale (SSD-12) was used to evaluate the psychological distress related to somatic symptoms. It consists of four items for each sub-criterion, representing cognition, emotion, and behavior [15,16]. SSD-12 scores range from 0 to 48; higher scores indicate greater distress. A cut-off score of ≥ 23 indicates psychological distress related to somatic symptoms. The SSD-12 was shown to have good item characteristics and excellent reliability (Cronbach α = .95) [16].

The functioning of the patients’ families was assessed using the Systemic Clinical Outcome and Routine Evaluation (SCORE-15) [17,18]. The SCORE-15 includes three subscales referring to perceptions of”strengths and adaptability,” “being overwhelmed by difficulties,” and “disrupted communication” within the family. For each item, answers are scored from 1 (“strongly agree”) to 5 (“strongly disagree”). A higher score indicates greater perceived difficulties in family functioning. The following cut-offs define clinical significance: 2.72 for the overall SCORE-15, 2.53 for the strengths subscale, 2.71 for the difficulties subscale, and 2.74 for the communication subscale [19].

At the beginning of the transition process the patient´s disease-specific knowledge and need for counseling were assessed using a transition questionnaire which was developed in the national project TRANSLATE NAMSE [7]. The questionnaire comprises 30 items addressing disease-specific knowledge concerning diagnosis, therapy, monitoring, and behavior management. Non-disease-specific items included the perceived level of independence when navigating the medical system, as well as the need for genetic, legal, and psychological counseling, as well as career options. Item responses were coded as follows: 3 = agree; 2 = partially agree; 1 = disagree; 0 = not relevant.

### Semi-structured interview

An interview guide was developed for qualitative data collection between January and April 2023. The interview was based on the findings of the anthology “When Children and Adolescents Have Chronic Physical Illnesses: Psychological and Social Development, Prevention, and Intervention”, edited by Martin Pinquart [20].

The aim of this interview was to gain insights into the psychosocial situation of the adolescents, to identify problems of their lives, and to collect information about possible support needs.

Therefore, the interview guide covered eight topics. First, general information about the illness, the symptoms, and the medical treatment is collected, followed by questions about emotional needs. The third section addresses the family situation, asking about the relationship with the parents, autonomy, and support needs. The section on school and friends focuses on challenges in an educational or work context and social perception. The fifth section explores the individual burden related to legal and financial needs. Furthermore, information is collected on experiences with medical care, specifically regarding the patient-provider relationship, appointment coordination, and the consideration of psychosocial concerns. The next section addresses the transition to adult medical care, including associated fears and need for information. The last two sections deal with structural problems during transition. The final part contains open-ended questions about needed support services and concerns that have not yet been addressed.

A total of 46 adolescents who scored high on specific questionnaires (PHQ-D > 10, GAD-7 > 5, SSS-8 > 8) were invited to participate in the interviews. A total of 17 adolescents initially agreed to participate, but three of them declined later. Semi-structured interviews were conducted with 14 adolescents between April and June 2023 to explore in more detail the specific reasons for the elevated psychosomatic burden, depression, and anxiety disorders. With the consent of the participants and their parents or stepparents, the interviews were audio recorded, transcribed, anonymized, and analysed using qualitative content analysis as described by Kuckartz [21].

### Qualitative content analysis

The transcribed semi-structured interviews of 14 participants were subsequently submitted to qualitative content analysis according to Kuckartz [21]. This method provides a systematic yet flexible framework for analysing qualitative data through the inductive development of categories while allowing new themes to emerge from the material [22]. Kuckartz’s method is closely modelled after Mayring’s qualitative content analysis, which combines adherence to rules with methodological verifiability, but also offers greater flexibility [23]. In contrast to Mayring, whose category system is comparatively rigid and oriented towards quantitative methods, Kuckartz’s approach offers greater versatility. This is important for extracting all relevant information from the material and remaining open to unexpected data [24].

The qualitative content analysis was aided by MAXQDA 2024 analytical software for data management and coding of qualitative interview data and addressed the following research question: In which areas do young people experience burden indicating that support is needed?

The analysis was performed by two researchers in iterative coding cycles. First, both coders familiarized themselves with the interview transcripts through repeated reading, highlighting relevant passages and recording initial analytical notes. Investigator triangulation was applied. Based on the coders’ observations and theoretical considerations, a tentative combined deductive-inductive system of main categories was developed and tested in a pilot coding to evaluate the applicability and clarity of the coding framework. The category system was subsequently refined through discussions within the research team. During the first coding cycle, relevant text segments were assigned to one or more of the previously developed main categories. Subsequently, subcategories were developed inductively within each main category and the complete dataset was recoded using the revised coding framework. Throughout the analysis, the coding system was continuously refined where necessary.

Finally, the findings were synthesized thematically for each category and systematically documented. The final coding framework comprised four inductively derived main categories: somatic complaints, psychological aspects, informational aspects, and structural problems. Within these categories, subcategories were developed inductively to capture more specific areas of burden and support needs identified in the interviews.

### Statistical analysis

Descriptive statistics were calculated for all questionnaire scores, for the total sample and separately for the four diagnostic subgroups. Percentages between groups were compared using χ^2^-tests. ANOVAs were used to compare the subgroups regarding psychosomatic symptom severity. Pearson’s correlation coefficients were calculated to estimate correlations between the transition questionnaire subscores and other variables. All analyses were performed using IBM SPSS Statistics for Windows, Version 29.0 (IBM Corp., Armonk, NY).

### Ethical Aspect of Research

The study was approved by the Ethics Committee of the University Hospital of Heidelberg (S-412/2022). Written informed consent was obtained from all parents/guardians, with assent from patients when appropriate for their age. Inclusion criteria were: (i) chronic disease, (ii) age ≥ 16 years, (iii) willingness to participate in a structured transition programme. Exclusion criteria were: (i) severe intellectual disability and (ii) lack of informed consent.

## Results

### Patient characteristics

Patient characteristics and diagnoses are shown in Table 1 and Supplemental Table 1. Fourty-six patients of the 92 (50%) participants suffered from gastrointestinal diseases, 16 (17.4%) from nephrological diseases, 21 (22.8%) from endocrine diseases, and 9 (9.8%) had other diagnoses. The “others” category includes six patients with cystic fibrosis, two with psoriatic arthritis, and one patient with Brachyolmia type 4 (Table 1). Most of the patients were high school students, followed by ”others“, which included university students and those doing a voluntary social year (Supplemental Table 1). The median age at enrollment was 17.95 years (range 16.02–24.21).

**Table 1:** Patient characteristics.

	Entire cohort	Patients with gastrointestinal diseases	Patients with nephrological diseases	Patients with endocrinological diseases	others
Number	92	46	16	21	9
Age (years) Range Median	17.95 ± 1.34 16.0 - 24.2 17.9	17.81 ± 1.18 16.0–20.5 17.8	18.98 ± 1.89 16.6–24.2 18.5	17.29 ± 1.35 16.4 - 20.1 17.6	16.29 ± 0.70 16.3–18.4 17.8
Female (%)	37 (40.2%)	22 (47.8%)	9 (56.3%)	4 (19.0%)	2 (22.2%)

Data are mean ± SD, if not indicated otherwise

### Psychosomatic Burden

Mean values and further statistics of the questionnaires measuring the psychosomatic burden are shown in Table 2. No significant differences were found between the diagnostic subgroups.

**Table 2:** Psycho-somatic characteristics of the study sample.

	Entire cohort (n = 92)	Patients with gastrointestinal diseases (n = 46)	Patients with nephrological diseases (n = 16)	Patients with endocrinological diseases (n = 21)	Others (n = 9)	Test statistic ^#^	
						F (3.88)	*P*-value
SSS-8 Score Mean (SD)	6.00 (4.72)	6.22 (4.44)	8.06 (5.86)	3.86 (3.62)	6.25 (5.01)	2.63	.055
Impairment due to somatic complaints (SSS-8 cutoff ≥8) n (%) [95% CI]	36 (39.6%) [29% - 50%]	19 (41.3%) [28% - 56%]	9 (56.3%) [29% - 82%]	6 (28.6%) [10% - 48%]	2 (25.0%) [0% - 60%]	χ^2^ (3) = 3.69, p = .297	
SSD-12 Score Mean (SD)	10.77 (8.22)	10.98 (6.76)	11.00 (5.99)	9.45 (10.44)	12.33 (12.81)	0.289	.833
Somatic stress disorder (SSD-12 cutoff ≥23) n (%) [95% CI]	6 (6.5%) [0% - 12%]	2 (4.3%) [0% - 11%]	0	3 (14.3%) [0% - 31%]	1 (11.1%) [0% - 40%]	χ^2^ (3) = 3.86, p = .277	
GAD-7 Score Mean (SD)	4.23 (3.92)	4.28 (3.59)	4.50 (3.22)	3.52 (3.83)	5.11 (6.58)	0.399	.754
mild generalized anxiety disorder (GAD-7: 5–9) n (%) [95% CI]	33 (35.9%) [26% - 46%]	19 (41.3%) [27% - 56%]	7 (43.8%) [20% - 70%]	5 (23.8%) [7% - 43%]	2 (22.2%) [0% - 40%]	χ^2^ (3) = 3.08, p = .380	
moderate generalized anxiety disorder (GAD-7: 10–14) n (%) [95% CI]	8 (8.7%) [3% - 15%]	3 (6.5%) [0% - 14%]	1 (6.3%) [0% - 21%]	2 (9.5%) [0% - 23%]	2 (22.2%) [0% - 40%]	χ^2^ (3) = 2.48, p = .478	
severe generalized anxiety disorder (GAD-7: ≥ 15) n (%) [95% CI]	3 (3.3%) [0% - 8%]	1 (2.2%) [0% - 7%]	0	1 (4.8%) [0% - 17%]	1 (11.1%) [0% - 40%]	χ^2^ (3) = 2.62, p = .454	
PHQ-9 Score Mean (SD)	5.35 (4.26)	5.35 (3.92)	6.25 (4.75)	4.52 (3.83)	5.67 (6.80)	0.510	.677
mild depression (PHQ-9: 5–9) n (%) [95% CI]	26 (28.3%) [20% - 38%]	11 (23.9%) [12% - 37%]	6 (37.5%) [13% - 62%]	7 (33.3%) [14% - 55%]	2 (22.2%) [0% - 63%]	χ^2^ (3) = 1.53, p = .675	
moderate depression (PHQ-9: 10–14) n (%) [95% CI]	15 (16.3%) [10% - 24%]	8 (17.4%) [7% - 29%]	3 (18.8%) [0% - 41%]	2 (9.5%) [0% - 23%]	2 (22.2%) [0% - 40%]	χ^2^ (3) = 1.05, p = .790	
severe depression (PHQ-9: *≥* 15) n (%) [95% CI]	4 (4.3%) [1% - 9%]	2 (4.3%) [0% - 12%]	1 (6.3%) [0% - 21%]	0	1 (11.1%) [0% - 40%]	χ^2^ (3) = 2.08, p = .555	
Score 15 Total score Mean (SD)	1.69 (0.63)	1.73 (0.70)	1.55 (0.35)	1.68 (0.62)	1.73 (0.74)	0.333	.801
Score-15 Strengths & adaptability Mean (SD)	1.69 (0.69)	1.77 (0.74)	1.45 (0.44)	1.69 (0.76)	1.69 (0.66)	0.862	.464
Score 15 Overload in case of difficulties Mean (SD)	1.64 (0.76)	1.64 (0.78)	1.54 (0.57)	1.71 (0.75)	1.69 (1.04)	0.171	.016
Score 15 Disturbed communication Mean (SD)	1.73 (0.71)	1.78 (0.81)	1.66 (0.56)	1.64 (0.59)	1.80 (0.71)	0.262	.853

^#^Difference between the four diagnostic groups according to ANOVA

Data are mean ± SD, if not indicated otherwise.

Somatic Symptom Scale – 8 (SSS-8); Somatic Symptom Disorder Scale-12 (SSD-12); Generalized Anxiety Disorder Scale-7 (GAD-7); Patient Health Questionnaire (PHQ-9); Systemic Clinical Outcome and Routine Evaluation (SCORE-15)

According to the SSS-8, 39.6% (95% CI = 29%, 50%) of the adolescents were moderately impaired by somatic complaints (Table 2). However, according to the SSD-12, only 6.5% (95% CI = 0%, 12%) of the sample raised severe concerns due to somatic symptoms (Table 2). 20.6% (95% CI = 10.3%, 28%) of the patients showed symptoms of a moderate or severe depression, whereas 11.9% (95% CI = 6.8%, 17.5%) of the adolescents suffered from moderate or severe generalized anxiety disorder symptoms (Table 2).

Table 2 shows the mean values for the self-assessment of family functioning (SCORE-15). The mean values for all three SCORE-15 subscales were low, indicating good family functioning overall. Correlation analyses revealed that, in the total sample, all SCORE-15 subscales and the total score were significantly correlated with psychosomatic symptom severity. Lower family functioning was associated with higher symptom severity for the SSS-8, SSD-12, PHQ-9, and GAD-7 (Supplemental Table 2).

### Transition Questionnaire

The results from the transition questionnaire are shown in Supplemental Table 3. Overall, knowledge about the disease and treatment was satisfactory. However, knowledge about future and career plans was low, and adolescents expressed the need for more support and information. No significant differences in knowledge about the disease, medication, or specific treatment were found among the different diagnostic groups. The mean score of each subscale ranged from 1.85 ± 0.76 for the “plans for the future and career”-subscale to 2.5 ± 0.42 for the “clinic contacts”-subscale. Correlation coefficients between the transition questionnaire and other variables are shown in Table 3.

**Table 3:** Correlation of questionnaires for psychosomatic burden with dimensions *therapy and treatment* and *clinical contacts* of the transition questionnaire.

	Entire cohort (n=49)		Patients with endocrinological diseases (n=14)		Patients with gastrointestinal diseases (n = 18)	
Correlations with dimension *therapy and treatment*						
	*r*	*P*	*r*	*P*	*r*	*P*
SSS-8	0.01	0.93	−0.35	0.22	0.12	0.64
SSD-12	−0.4	**0.004**	−0.76	**0.002**	−0.10	0.69
GAD-7	−0.37	**0.009**	−0.065	**0.012**	0.15	0.56
PHQ-9	−0.11	0.43	−0.51	0.06	0.21	0.40
Score 15 (Total score)	−0.41	**0.004**	−0.62	**0.017**	−0.02	0.56
Score-15 1 (Strengths & adaptability)	−0.34	**0.016**	−0.52	0.059	−0.18	0.49
Score 15 2 (Overload in case of difficulties)	−0.4	**0.005**	−0.6	**0.022**	−0.02	0.93
Score 15 3 (Disturbed communication)	−0.29	**0.042**	−0.54	**0.046**	0.08	0.75
Correlations with dimension *clinical contacts*						
	*r*	*P*	*r*	*P*	*r*	*P*
SSS-8	0.36	**0.013**	0.49	0.08	0.53	**0.024**
SSD-12	0.23	0.11	0.63	**0.015**	0.30	0.23
GAD-7	0.22	0.14	0.26	0.38	0.33	0.18
PHQ-9	0.19	0.18	0.28	0.34	0.22	0.37
Score 15 (Total score)	0.17	0.26	0.47	0.09	0.02	0.96
Score-15 1 (Strengths & adaptability)	0.16	0.26	0.43	0.13	−0.05	0.14
Score 15 2 (Overload in case of difficulties)	0.15	0.32	0.41	0.14	−0.01	0.98
Score 15 3 (Disturbed communication)	0.11	0.46	0.41	0.14	0.09	0.72

A significant negative correlation was found between the “therapy and treatment”-scale and the SSD-12 (r = −0.4; p = 0.004) and GAD-7 (r = −0.37; p = 0.009). This suggests that lower knowledge of therapy and treatment is associated with higher psychological distress and anxiety related to somatic symptoms. This scale also correlated significantly negatively with the total SCORE-15 score (r = −0.41; P = 0.004), indicating that a worse self-perceived family functioning was associated with lower knowledge of therapy and treatment. The correlation was significant across all SCORE-15 subscales (see Table 3).

The dimension “clinical contacts” of the transition questionnaire correlated positively with the SSS-8 score (r = 0.36; P = 0.013), indicating that patients who independently manage their appointments experience greater impairment from somatic complaints. The correlation of the transition questionnaires for patients with endocrinological and gastrointestinal diseases are shown in Table 3. The anonymized data set of all Questionnaires is included in Supplemental Table 4.

### Semi-structured interview

Four main categories were inductively derived from the qualitative analysis of the interviews. These categories were “somatic complaints”, “psychological aspects”, “informative aspects”, and “structural problems”.

**Somatic complaints**: Regarding somatic complaints, the interviewees stated that they were primarily burdened by “loss of performance due to illness”. These complaints were both psychological, including concentration problems, and physical.

*“Like I said, I often feel limited at school. And I sometimes get the sense that this might prevent me from performing as well as I’d like to.” (Interview 10, pos. 68)*

**Psychological aspects:** Eight distinct factors were found to contribute to psychological distress: “chronic disease”, “psychological comorbidity”, “family”, “autonomy”, “fear of the future”, “school and work”, “friends”, and “medical staff”. In the “family”-subcategory, interviewees reported family stress caused by the young person’s illness, such as increased workload or conflicts, as well as stress experienced by the young person due to family dynamics.

*“It’s not just about me—my mom is also affected. For example, she sometimes has to drive me places or pick me up from school when I’m not feeling well. That’s caused issues at her work from time to time, like conflicts with her boss.“ (Interview 10, pos. 62)*

Regarding “autonomy”, interviewees noted a lack of competence related to their personal development and medical care.

*“I try to handle most of it on my own. But the problem is that my mom is still very attached to me—I wouldn’t say she’s clingy, but she still feels like she has to keep an eye on everything. It’s really hard for her to let go.” (Interview 14, pos. 50)*

In the category “fears about the future”, interviewees described concerns related to their chronic illness, such as a worsening of their condition. Additionally, they expressed fears about the future, including financial insecurity and restricted job prospects resulting from their illness.

*“I’m always worried about what my future will look like—how much I’ll earn, how much I’ll be able to save. And whether things will really stay stable, or if there will be difficult times ahead. That’s probably my biggest concern.” (Interview 4, pos. 94)*

Negative experiences at school or at work (e.g., pressure to perform, bullying, and conflicts), with friends or peers or with medical staff also contribute to the psychological stress experienced by young people.

**Informational aspects:** Interviewees described both “information deficits in themselves” and “information deficits in others” (e.g., parents, teachers, and society). This lack of “information for young people” included topics such as career, school, medical aspects, and transition. Several interviewees expressed a need for more information, particularly about the transition process.

*“Yeah. I felt like the doctors did talk to me about it, but it was all pretty general. I think if they had gone into more detail about how everything would actually work, I might have felt a bit more secure.” (Interview 10, pos. 118)*

**Structural problems**: Interviewees reported experiencing stress related to “clinic visits”, “work”, “school”, and insufficient “financial support”. In the context of clinic visits, poor infrastructure, such as long distances to the clinic, and difficulties scheduling appointments were described. Specific issues included frequent changes of physicians and long waiting times. With regard to “work” and “school”, participants reported problems resulting from frequent absences due to illness and the need to attend medical appointments.

*“I had already started a vocational training program back then, but unfortunately I had to drop out because I was absent too often—mainly due to illness or because I just wasn’t feeling well.” (Interview 13, pos. 90)*

One interviewee mentioned feeling burdened by a lack of financial support, such as a care allowance.

## Discussion

This is the first study to investigate family functioning and psychosocial burden in adolescents and young adults with chronic illnesses at the beginning of the transition period.

### Psychosomatic Characteristics

The assessment of psychosomatic burden revealed that the prevalence rate of moderate or severe generalized anxiety symptoms in our sample was 11.9% (95% CI = 6.8%, 17.5%), which is markedly higher than the 5.5% prevalence rate in healthy young people [25].

However, this rate is comparable to those found in children and adolescents with inflammatory bowel disease [26]. Similarly, the prevalence rate of 20.6% (95% CI = 10.3%, 28%) for moderate or severe depressive symptoms in our study population was markedly higher than population-based rates in young people which vary between 3.5% and 8.1% [25,27]. However, the rate we observed is comparable to the rate of 25.2% found in children and adolescents with inflammatory bowel disease, but much higher than in those with chronic kidney disease [28,29]. It is important to evaluate psychiatric disorders in adolescents with chronic diseases in order to initiate treatment as soon as possible. We therefore propose that patients scoring ≥ 10 on the GAD-7 should receive an extended diagnostic evaluation for anxiety disorders followed by appropriate treatment [30].

### Family functioning

Our results show that adolescents with chronic illnesses predominantly perceive the three dimensions of family functioning (i.e., strengths and adaptability, overload in the event of difficulties, and disturbed communication) favourably. To our knowledge, there are only two other studies that have assessed family functioning in a pediatric patient sample [31]. Fay et al. also found positive family assessments: Using a scoring system ranging from 1 to 6 for the total SCORE-15, it was shown that 46.2% of pediatric patients scored below 2, while only 11.8% scored above 3 [31], indicating that chronically-ill young people largely view their families as helpful and functional. This likely reflects the substantial support families with chronically-ill children provide to facilitate both medical care and social inclusion. The second study evaluated family functioning using the SCORE-15 in families with severely chronically-ill children experiencing a high level of psychosocial stress [4]. The children (median age of 13 years) reported no family dysfunction; only 14.3% of their parents reported reduced family functioning}. Interestingly, all of the SCORE-15 subscales were significantly associated with the severity of depression, anxiety, and somatic symptoms. A lower assessment of family functioning was associated with a higher psychosomatic burden in adolescents and young adults. The positive correlation between the “overload in case of difficulties”-dimension and anxiety severity suggests that adolescents feel more fearful and insecure when their families cannot cope with conflicts. This highlights the importance of family members providing support to chronically ill adolescents in order to build resilience and secure attachment in children. Based on these results, families experiencing high psychosocial stress warrant referral to child and adolescent psychosomatic or psychiatric specialists for treatment [4].

### Knowledge of illness and therapy

We identified a significant negative correlation between knowledge of therapy and treatment recorded in the transition questionnaire and the SSD-12 and total SCORE-15 scores. These results imply that adolescents with limited knowledge of their medical care may experience greater impairment from somatic symptoms and heightened anxiety, particularly within the context of problematic family dynamics.

### Support needs

A recent study of adolescents during the transition period revealed that only 7.3% of those with chronic diseases and 14% of those with chronic kidney disease requested psychological counseling. This contradicts the high frequency (72%) of psychological counseling provided, especially to kidney transplant recipients [7,32]. This discrepancy can be explained by the denial of psychological problems during adolescence, a challenging life stage characterized by a reduced likelihood of seeking help [33]. These findings align with our data collected during the semi-quantitative interviews. Qualitative content analysis of the interviews revealed impairment in all investigated categories and provided a variety of explanations for the elevated psychosomatic burden in individual cases. For example, adolescents were worried deeply about their future, which could explain the increased general anxiety scores. Another person vividly described the ‘vicious cycle` between somatic and psychological burden, which gives an explanation for the elevated somatic symptom scores. Interestingly, the quantitative analysis showed a good average family functioning. However, looking deeper in personal reports, qualitative analysis revealed delayed autonomy movements or separation from the parents in some cases. The individual statements in the interview demonstrated that adolescents with chronic illnesses often rely on their parents’ support, which contrasts with their natural desire for independence.

In a recent study, results from the transition questionnaire showed that even when the patients described their problems and fears in detail, they did not ask for help or support [32]. In order to address this issue and to support chronically ill patients during their transition, the evaluation of psychosocial stress should be implemented in the transition program. Furthermore, we strongly recommend offering active psychosocial support repeatedly before transfer is scheduled. An appropriate intervention of adaptation for adolescents with chronic diseases could be the Roy Adaptation Model in clinical nursing care to improve adaptation responses in psychological, self-concept, role function, and independence modes [34]. For example, in a young adolescent diagnosed with type 1 diabetes, it was demonstrated that the use of the Roy Adaptation Model was appropriate and beneficial, considering that the most common problems among adolescents hospitalized for T1D are physical, psychological, and psychosocial adaptation to the illness [35].

To the best of our knowledge, this study represents the most extensive and systematic examination of psychosomatic symptom burden and family functioning among adolescents with chronic illness throughout the transition phase. One strength of our study is the integration of validated questionnaires and semi-structured interviews at the beginning of the established transition process. However, the study is limited by its small sample size, particularly in the subgroup analyses. Additionally, as a single-center study, it does not cover all possible psychosocial factors, such as family conflicts, which may influence outcomes. Another limitation is that the anxiety and depression disorders were assessed solely using questionnaires without clinical confirmation of diagnoses. We invited adolescents who scored high on the specific questionnaires to participate in the interview. Therefore, we could miss the needs for support in adolescents with lower psychosomatic burden. Further research is warranted to evaluate whether our findings can be generalized to adolescents with other chronic diseases.

## Conclusion

This study indicates that adolescents with chronic diseases experience a psychosomatic burden at the beginning of the transition period, which is associated with lower family functioning. We found that a lack of knowledge about therapy and treatment is associated with somatic stress disorders and anxiety. Consequently, comprehensive education and systematic assessment of psychosocial burden and family functioning should be routinely embedded within transition pathways. We recommend that the pediatrician should repeatedly offer specific support and initiate early interventions, if necessary, that are likely to improve the course of the disease as well as the patient´s overall well-being before transfer is scheduled.

## Supporting information

S1 Table Patient characteristics. (DOCX)

S2 Table Pearson-correlation of SCORE-15 with psychosocial questionnaires (n = 92). (DOCX)

S3 Table Transition Questionnaire. (DOCX)

S4 Table Anonymized data set of all Questionnaires. (XLSX)

## References

[1] Lange KEG. Psychologische und pädagogische Elemente der Langzeitbehandlung bei Kindern und Jugendlichen mit chronischen Erkrankungen. 2 ed. Hiort O, Wabitsch M, editors. Berlin/Heidelberg: Springer. 2019.

[2] Hölling H, Schlack R, Kamtsiuris P, Butschalowsky H, Schlaud M, Kurth BM. The KiGGS study. Nationwide representative longitudinal and cross-sectional study on the health of children and adolescents within the framework of health monitoring at the Robert Koch Institute. Bundesgesundheitsblatt Gesundheitsforschung Gesundheitsschutz. 2012;55(6–7):836–42. doi: 10.1007/s00103-012-1486-3 22736165

[3] Arlt W, Willis DS, Wild SH, Krone N, Doherty EJ, Hahner S, et al. Health status of adults with congenital adrenal hyperplasia: a cohort study of 203 patients. J Clin Endocrinol Metab. 2010;95(11):5110–21. doi: 10.1210/jc.2010-0917 20719839 PMC3066446

[4] Zumbaum-Fischer F, Hartmann M, Kotzaeridou U, Mondry A, Resch F, Hoffmann GF, et al. The Interdisciplinary Family Consultation in Pediatrics - An Integrative Approach to Complex Burdened Families of Chronically ill Children. Psychother Psychosom Med Psychol. 2023;73(12):510–5. doi: 10.1055/a-2136-7397 37709285

[5] Blum RW, Garell D, Hodgman CH, Jorissen TW, Okinow NA, Orr DP, et al. Transition from child-centered to adult health-care systems for adolescents with chronic conditions. A position paper of the Society for Adolescent Medicine. J Adolesc Health. 1993;14(7):570–6. doi: 10.1016/1054-139x(93)90143-d 8312295

[6] Sawyer SM, Drew S, Yeo MS, Britto MT. Adolescents with a chronic condition: Challenges living, challenges treating. Lancet. 2007;369(9571):1481–9. doi: 10.1016/S0140-6736(07)60370-5 17467519

[7] Grasemann C, Höppner J, Burgard P, Schündeln MM, Matar N, Müller G, et al. Transition for adolescents with a rare disease: Results of a nationwide German project. Orphanet J Rare Dis. 2023;18(1):93. doi: 10.1186/s13023-023-02698-2 37098531 PMC10131406

[8] Pelentsov LJ, Laws TA, Esterman AJ. The supportive care needs of parents caring for a child with a rare disease: A scoping review. Disabil Health J. 2015;8(4):475–91. doi: 10.1016/j.dhjo.2015.03.009 25959710

[9] Nibras S, Kentor R, Masood Y, Price K, Schneider NM, Tenenbaum RB, et al. Psychological and psychiatric comorbidities in youth with serious physical illness. Children (Basel). 2022;9(7):1051. doi: 10.3390/children9071051 35884035 PMC9316756

[10] Kroenke K, Spitzer RL, Williams JB. The PHQ-9: Validity of a brief depression severity measure. J Gen Intern Med. 2001;16(9):606–13. doi: 10.1046/j.1525-1497.2001.016009606.x 11556941 PMC1495268

[11] Löwe B, Spitzer RL, Gräfe K, Kroenke K, Quenter A, Zipfel S, et al. Comparative validity of three screening questionnaires for DSM-IV depressive disorders and physicians’ diagnoses. J Affect Disord. 2004;78(2):131–40. doi: 10.1016/s0165-0327(02)00237-9 14706723

[12] Spitzer RL, Kroenke K, Williams JBW, Löwe B. A brief measure for assessing generalized anxiety disorder: the GAD-7. Arch Intern Med. 2006;166(10):1092–7. doi: 10.1001/archinte.166.10.1092 16717171

[13] Gierk B, Kohlmann S, Kroenke K, Spangenberg L, Zenger M, Brähler E, et al. The somatic symptom scale-8 (SSS-8): A brief measure of somatic symptom burden. JAMA Intern Med. 2014;174(3):399–407. doi: 10.1001/jamainternmed.2013.12179 24276929

[14] Kroenke K, Jackson JL, Chamberlin J. Depressive and anxiety disorders in patients presenting with physical complaints: clinical predictors and outcome. Am J Med. 1997;103(5):339–47. doi: 10.1016/s0002-9343(97)00241-6 9375700

[15] Kop WJ, Toussaint A, Mols F, Löwe B. Somatic symptom disorder in the general population: Associations with medical status and health care utilization using the SSD-12. Gen Hosp Psychiatry. 2019;56:36–41. doi: 10.1016/j.genhosppsych.2018.10.004 30578985

[16] Toussaint A, Murray AM, Voigt K, Herzog A, Gierk B, Kroenke K, et al. Development and Validation of the Somatic Symptom Disorder-B Criteria Scale (SSD-12). Psychosom Med. 2016;78(1):5–12. doi: 10.1097/PSY.0000000000000240 26461855

[17] Stratton P, Lask J, Bland J, Nowotny E, Evans C, Singh R, et al. Detecting therapeutic improvement early in therapy: validation of the SCORE ‐15 index of family functioning and change. Journal of Family Therapy. 2013;36(1):3–19. doi: 10.1111/1467-6427.12022

[18] Stratton P, Bland J, Janes E, Lask J. Developing an indicator of family function and a practicable outcome measure for systemic family and couple therapy: the SCORE. Journal of Family Therapy. 2010;32(3):232–58. doi: 10.1111/j.1467-6427.2010.00507.x

[19] Miller RB, Nebeker-Adams CA, Anderson SR, Bradford AB, Johnson LN. The development of a reliable change index and cutoff score for the SCORE-15. J Marital Fam Ther. 2023;49(1):36–48. doi: 10.1111/jmft.12600 35913047

[20] Pinquart M. Wenn Kinder und Jugendliche körperlich chronisch krank sind. 1 ed. Heidelberg: Springer Berlin. 2013.

[21] Kuckartz U. Qualitative Inhaltsanalyse. Methoden, Praxis, Computerunterstützung. Weinheim Basel: Beltz Verlag. 2016.

[22] Kuckartz U. Qualitative Inhaltsanalyse. Methoden, Praxis, Computerunterstützung. Beltz Verlag2022. 2022. 135–56 p.

[23] Kuckartz U. Qualitative Inhaltsanalyse. Methoden, Praxis, Computerunterstützung. Weinheim: Beltz Verlag. 2018.

[24] Kuckartz U. Qualitative Inhaltsanalyse. Methoden, Praxis, Computerunterstützung. Beltz Verlag. 2022.

[25] Organization WH. Mental health of adolescents. https://www.who.int/news-room/fact-sheets/detail/adolescent-mental-health. Accessed 2025 July 17.

[26] Stapersma L, van den Brink G, Szigethy EM, Escher JC, Utens EMWJ. Systematic review with meta-analysis: anxiety and depression in children and adolescents with inflammatory bowel disease. Aliment Pharmacol Ther. 2018;48(5):496–506. doi: 10.1111/apt.14865 29984495

[27] Shorey S, Ng ED, Wong CHJ. Global prevalence of depression and elevated depressive symptoms among adolescents: A systematic review and meta-analysis. Br J Clin Psychol. 2022;61(2):287–305. doi: 10.1111/bjc.12333 34569066

[28] Barberio B, Zamani M, Black CJ, Savarino EV, Ford AC. Prevalence of symptoms of anxiety and depression in patients with inflammatory bowel disease: A systematic review and meta-analysis. Lancet Gastroenterol Hepatol. 2021;6(5):359–70. doi: 10.1016/S2468-1253(21)00014-5 33721557

[29] Kogon AJ, Matheson MB, Flynn JT, Gerson AC, Warady BA, Furth SL, et al. Depressive Symptoms in Children with Chronic Kidney Disease. J Pediatr. 2016;168:164-170.e1. doi: 10.1016/j.jpeds.2015.09.040 26505290 PMC4854431

[30] Löwe B, Decker O, Müller S, Brähler E, Schellberg D, Herzog W, et al. Validation and standardization of the generalized anxiety disorder screener (GAD-7) in the general population. Med Care. 2008;46(3):266–74. doi: 10.1097/MLR.0b013e318160d093 18388841

[31] Fay D, Carr A, O’Reilly K, Cahill P, Dooley B, Guerin S, et al. Irish norms for the SCORE‐15 and 28 from a national telephone survey. Journal of Family Therapy. 2011;35(1):24–42. doi: 10.1111/j.1467-6427.2011.00575.x

[32] Choukair D, Rieger S, Bethe D, Treiber D, Hoffmann GF, Grasemann C, et al. Resource use and costs of transitioning from pediatric to adult care for patients with chronic kidney disease. Pediatr Nephrol. 2024;39(1):251–60. doi: 10.1007/s00467-023-06075-w 37464057 PMC10673743

[33] Wilson CJ, Deane FP, Marshall KL, Dalley A. Adolescents’ suicidal thinking and reluctance to consult general medical practitioners. J Youth Adolesc. 2010;39(4):343–56. doi: 10.1007/s10964-009-9436-6 20229227

[34] Roy C, Whetsell MV, Frederickson K. The Roy adaptation model and research. Nurs Sci Q. 2009;22(3):209–11. doi: 10.1177/0894318409338692 19694080

[35] Güneş EO, Cetinkaya S. The use of the Roy Adaptation Model in clinical nursing care of an adolescent diagnosed with type 1 diabetes (case report). Front Clin Diabetes Healthc. 2026;7:1721420. doi: 10.3389/fcdhc.2026.1721420 42079479 PMC13129417

