# Supplementary material for: Psychosomatic symptom burden and family functioning of adolescents with chronic diseases in transition to adult medicine – Implications for psychosocial support
Source: PLoS One. 2026 Sep 30;21(9):e0359441. doi: 10.1371/journal.pone.0359441 (PMC13626331; doi:10.1371/journal.pone.0359441)
Supplement: Supplementary file 1 [file pone.0359441.s001.docx]

**Supplemental Table 1:** Patient characteristics

|  | Entire  cohort (N = 92) | Patients with gastrointestinal  diseases  (N = 46) | Patients with nephrological  diseases  (N = 16) | Patients with endocrinological  diseases (N = 21) | Others  (N = 9) |
| --- | --- | --- | --- | --- | --- |
| **Specific diagnoses** |  | - Colitis ulcerosa (*n* = 19) - M. Crohn *(n* = 17) - Liver transplant recipients *(n = 6)* - CIPO *(n=1)* - Autoimmune hepatitis *(n=1)* - Hepatopathy in cholestasis *(n=1)* - Stomach ache caused by intestinal transit disorder and multiple abdominal procedures *(n=1)* | - Kidney transplant recipients *(n= 12**)* - Nephrotic syndrome *(n=4*)* - Midaortic syndrome with renal artery stenosis *(n=1)* | - Diabetes mellitus type 1 *(n= 7)* - Growth hormone deficiency *(n= 5)* - Hypergonadotropic hypogonadism due to chromosomal abnormalities *(n= 2*) - Prolactinoma *(n=2)* - Panhypopituitarism due to germinoma *(n=2) and p*ilocytic astrocytoma *(n=1)* - Hypogonadotropic hypogonadism *(n=1)* - Primary hypothyroidism *(n=1)* | - Cystic fibrosis *(n= 6)* - Psoriatic arthritis *(n= 2)* - Brachyolmia type 4 *(n=1)* |
| **Education** |  |  |  |  |  |
| Special education school/ school for children with learning difficulties | 4 | 2 | 0 | 2 | 0 |
| Secondary school with the lowest school leaving qualification/certificate | 1 | 1 | 0 | 0 | 0 |
| Secondary school with the intermediate school leaving qualification/certificate | 8 | 3 | 2 | 1 | 2 |
| School in which different educational programs are offered at one and the same school | 2 | 1 | 1 | 0 | 0 |
| Secondary school with the highest school leaving qualification/certificate | 36 | 20 | 3 | 10 | 3 |
| Vocational school | 14 | 5 | 3 | 4 | 2 |
| Others | 27 | 14 | 7 | 4 | 2 |

Data are mean ± SD, if not indicated otherwise.

* Patient with minimal change disease and colitis ulcerosa (here counted to nephrotic syndrome)
** Patient with combined kidney- and liver transplantation (here counted to kidney transplant recipient)
