# Supplementary material for: Psychosomatic symptom burden and family functioning of adolescents with chronic diseases in transition to adult medicine – Implications for psychosocial support
Source: PLoS One. 2026 Sep 30;21(9):e0359441. doi: 10.1371/journal.pone.0359441 (PMC13626331; doi:10.1371/journal.pone.0359441)
Supplement: Supplementary file 2 [file pone.0359441.s002.docx]

**Supplemental Table 2:** Pearson-correlation of SCORE-15 with psychosocial questionnaires (n=92)

|  | SSS-8 | SSD-12 | GAD-7 | PHQ-9 |
| --- | --- | --- | --- | --- |
| Score 15  Total score | .373** | .499** | .589** | .451** |
| Score-15  Strengths & adaptability  Mean (SD) | .266* | .416** | .476** | .400** |
| Score 15 Overload in case of difficulties  Mean (SD) | .399** | .591** | .641** | .495** |
| Score 15  Disturbed communication Mean (SD) | .373* | .271** | .411** | .275** |

* p<0.05

** p<.001

Systemic Clinical Outcome and Routine Evaluation (SCORE-15); Somatic Symptom Scale – 8 (SSS-8); Somatic Symptom Disorder Scale-12 (SSD-12);

Generalized Anxiety Disorder Scale-7 (GAD-7); Patient Health Questionnaire (PHQ-9)
