# Supplementary material for: Psychosomatic symptom burden and family functioning of adolescents with chronic diseases in transition to adult medicine – Implications for psychosocial support
Source: PLoS One. 2026 Sep 30;21(9):e0359441. doi: 10.1371/journal.pone.0359441 (PMC13626331; doi:10.1371/journal.pone.0359441)
Supplement: Supplementary file 3 [file pone.0359441.s003.docx]

**Supplemental Table 3:** Transition Questionnaire

|  | Entire  cohort  (n=49) | Patients with gastrointestinal  Diseases (n=18) | Patients with nephrological  Diseases  (n=11) | Patients with endocrinological  Diseases (n=14) | Others  (n=6) | Test statistic ^#^ | |
| --- | --- | --- | --- | --- | --- | --- | --- |
|  |  |  |  |  |  | F (3,45) | *P*-value |
| Total Score  Mean (SD) | 2.16 (0.36) | 2.12 (0.30) | 2.33 (0.25) | 2.08 (0.50) | 2.14 (0.36) | 1.075 | .369 |
| Knowledge about the disease  Mean (SD) | 2.21 (0.52) | 2.27 (0.52) | 2.13 (0.44) | 2.09 (0.63) | 2.47 (0.37) | 0.903 | .447 |
| Therapy & treatment  Mean (SD) | 2.28 (0.47) | 2.25 (0.26) | 2.51 (0.19) | 2.27 (0.75) | 2.00 (0.43) | 1.639 | .194 |
| Support & Information  Mean (SD) | 1.95 (0.47) | 1.94 (0.45) | 2.09 (0.34) | 1.79 (0.53) | 2.05 (0.61) | 0.989 | .407 |
| Plans for the future & career  Mean (SD) | 1.85 (0.76) | 1.70 (0.62) | 2.27 (0.62) | 1.73 (0.95) | 1.83 (0.79) | 1.537 | .281 |
| Clinic contacts Mean (SD) | 2.50 (0.42) | 2.43 (0.50) | 2.64 (0.32) | 2.55 (0.41) | 2.33 (0.37) | 0.947 | .426 |

\# Difference between the four diagnostic groups according to ANOVA

Data are mean ± SD, if not indicated otherwise.
