# Supplementary material for: Psychosomatic symptom burden and family functioning of adolescents with chronic diseases in transition to adult medicine – Implications for psychosocial support
Source: PLoS One. 2026 Sep 30;21(9):e0359441. doi: 10.1371/journal.pone.0359441 (PMC13626331; doi:10.1371/journal.pone.0359441)
Supplement: Supplementary file 4 [file pone.0359441.s006.doc]

| Center for rare diseases - University Hospital Heidelberg Im Neuenheimer Feld 130.3 \| 69120 Heidelberg |  | **Center for rare diseases**  **University Hospital Heidelberg**  Chair:  Prof. Dr. med. Prof. h.c. mult (RCH)  Georg F. Hoffmann  PD Dr. med. Daniela Choukair  Medical coordinator  Im Neuenheimer Feld 130.3  69120 Heidelberg  Phone +49 (0)6221 56 4002 (information)  56 2396 (office)  56 32928 (extention)  fax +49 (0)6221 56 4501  Daniela.Choukair@  med.uni-heidelberg.de  [www.seltene-erkrankungen-heidelberg.de](http://www.seltene-erkrankungen-heidelberg.de/)  30.11.2026/ DC |
| --- | --- | --- |
| Univ.-Prof. Dr. Stephan Doering  Academic Editor  PLOS one  Leiter der Klinik für Psychoanalyse  und Psychotherapie  Medizinische Universität Wien  Währinger Gürtel 18-20  A-1090 Wien |  |  |
|  |  |  |

**Revision of the Manuscript PONE-D-25-54330: Psychosomatic Symptom Burden and Family Functioning of Adolescents with Chronic Diseases in Transition to Adult Medicine – Implications for Psychosocial Support**

Dear Prof. Dr. Doering, dear Editorial Board,

Enclosed please find the revision of the above manuscript.

We appreciate the reviewer’s careful evaluation of the manuscript and have addressed the comments in the revised manuscript. The changes made in the manuscript are highlighted. The data generated or analysed during this study are included in this published article and its supplementary information file (supplemental Table S4).

Please see below our detailed response to the reviewer’s concerns.

**Response to the comments of reviewer 1:**

While I do have some knowledge about adolescent mental health, the topic and context of this particular paper is outside my area of expertise. I am also not an expert in qualitative methods or research. I do hope that the editors have invited reviewers who know the filed better than me.

Having said that, I find the paper well-written, thorough, and mostly clear. My comments (in no particular order):

1. The perhaps most major comment concerns the mixed methods approach. While this is commendable, and provides opportunities for synergy effects, I think the approach is underdeveloped in this case. The two analyses mostly appear as parallel analyses, with very little cross-breeding. This is especially evident in the discussion-section, where the results of the qualitative analysis are only mentioned in two sentences (lines 373-375). Overall, the qualitative analysis is relatively more neglected and appears to be somewhat of a sideshow. I suggest greater integration between the quantitative and qualitative parts, or alternatively, dropping the qualitative one.

*We agree with the reviewer that the qualitative results were not adequately integrated into the discussion. In the revised manuscript we have inserted a paragraph to discuss the qualitative results and relate them to the results of the quantitative analysis (please see lines 415-428).*

2. Only adolescents who scored high on specific questionnaires (PHQ-D > 10, GAD-7 >5, SSS-8 > 8) were invited to participate in the interviews. What is the motivation for this selection strategy? What do the authors gain (or miss) from focusing on this group?

*We selected adolescents or young adults with a high score in depression, anxiety, or somatic symptom severity because we hypothesized that these persons would be most likely to talk about problem areas and describe their support needs. However, we agree with the reviewer that with this selection strategy we also missed out on the thoughts of less burdened chronically ill adolescents and young adults. We have now included this as a limitation of the interview study (lines 448-451).*

3. The description of the qualitative method is inadequate and insufficient. The method (Kuckhartz, which I don’t know anything about) is described in one sentence (lines 172-174). I can understand that specific qualitative methods are more difficult to describe in a general way (they are less standardized), but readers need more context here.

*We have now described the qualitative content analysis in more detail (please see methods section, lines 181-209).*

4. The connection between the four categories and the aim of “identifying psychosocial situations and contexts in which the adolescent experiences psychosocial stress” (lines 143-144) is not clear to me. This is particularly evident in relation to the “somatic complaints”- and “informational aspects”-categories: how do these categories address the aim? Likewise, the “psychological aspects”- and the ”structural problems”-categories seems to be about causes of psychosocial stress, not contexts. Moreover, the “somatic complaints”-category seems underdeveloped to me.

*We have now clarified the aim of the interview (lines 156-158). We also clarified the main research question that determined the qualitative content analysis (lines 192-193).*

5. The authors make a big deal about the “transition-framing” (e.g., “during the transition period”, “transition process”, “throughout the transition phase”). These terms, including transition itself, imply a temporal process: a move, in time, from one state to another. It is not clear to me in what way the design of study, given the cross-sectional data, really addresses this issue. It appears more as a snapshort of the situation at the beginning of a transition period. While this is also a valid topic, right now there is a discordance between the design and the framing of the study.

*It is correct that the study was cross-sectional, capturing one time point in the process of transition. We have now clarified this in the revised manuscript. We have also shortened the description of the transition process to underline that we focus on the beginning of the transition.*

Minor comments:

6. Tables 2 and 3 may be integrated into one for improved clarity and efficiency.

*We have integrated tables 2 and 3 into one table.*

7. There appears to be a few minor errors in the writing that the authors (or an LLM) can probably pick up with a close reading of the manuscript.

*We have now re-checked the manuscript for language errors .*

**Response to the comments of the reviewer 2:**

Dear author, first of all, I am very pleased to review your valuable work.

Major comments:

1. The objective and study design are not clearly stated in the abstract.

*We have inserted in the abstract the objective of the study (line 29-30): “The aim was to evaluate psychosocial burden, family functioning, knowledge of their illnesses and identify needs for support”*

The methodology section might be more structured if presented under headings like those below.

Study design

Research questions or Hypothesis

Data collection tools

Collection of Data

Statistical analysis

*We have restructured the methodology section according to the reviewer’s suggestion.*

2. In the measurement section, no information is provided regarding whether validity and reliability analyses were conducted for the scales or concerning the validity and reliability coefficients; it would be appropriate to include this information, citing the relevant references.

*We have now included information regarding reliability coefficients and validity of the questionnaires (lines 113-116; 121; 126-127; 132-133; 139-141).*

3. Since multiple scales are used in the study, it is recommended to identify the research questions individually and write the discussion section using headings based on these questions. This would allow for a more systematic discussion.

*We have now divided the discussion into sections and added different subheadings.*

4. Incorporating the results of recent intervention studies addressing anxiety, ineffective role performance, family-centered care, social interaction and lack of information about ilness in adolescents with chronic illnesses could enrich the discussion. You may utilize the results section of the recent example study provided below.

*We have inserted in the discussion a paragraph which addresses recent intervention studies (line 434-440): “An appropriate intervention of adaptation for adolescents with chronic diseases could be the Roy Adaptation Model in clinical nursing care to improve adaptation responses in psychological, self-concept, role function, and independence modes (34). For example, in a young adolescent diagnosed with type 1 diabetes, it was demonstrated that the use of the Roy Adaptation Model was appropriate and beneficial, considering that the most common problems among adolescents hospitalized for T1D are physical, psychological, and psychosocial adaptation to the illness (35).”*

After all comments have been addressed, we will be very happy if our manuscript can be considered for publication in PLOS One.

On behalf of all the authors,

Yours sincerely,

Daniela Choukair

Consultant Pediatric Endocrinology and Diabetology

Medical Coordinator of the Center for Rare Diseases
