# Supplementary material for: Psychosomatic symptom burden and family functioning of adolescents with chronic diseases in transition to adult medicine – Implications for psychosocial support
Source: PLoS One. 2026 Sep 30;21(9):e0359441. doi: 10.1371/journal.pone.0359441 (PMC13626331; doi:10.1371/journal.pone.0359441)
Supplement: Peer review history

# Peer review: Decision Letter 0

**DOI:** 10.1371/journal.pone.0359441.r001

**Authors:** Stephan Doering

15 Jul 2026

Dear Dr. Choukair,

Thank you for submitting your manuscript to PLOS ONE. After careful consideration, we feel that it has merit but does not fully meet PLOS ONE’s publication criteria as it currently stands. Therefore, we invite you to submit a revised version of the manuscript that addresses the points raised during the review process.

Please submit your revised manuscript by Aug 29 2026 11:59PM. If you will need more time than this to complete your revisions, please reply to this message or contact the journal office at plosone@plos.org. When you're ready to submit your revision, log on to https://www.editorialmanager.com/pone/ and select the 'Submissions Needing Revision' folder to locate your manuscript file.

- A letter that responds to each point raised by the academic editor and reviewer(s). You should upload this letter as a separate file labeled 'Response to Reviewers'.
- A marked-up copy of your manuscript that highlights changes made to the original version. You should upload this as a separate file labeled 'Revised Manuscript with Track Changes'.
- An unmarked version of your revised paper without tracked changes. You should upload this as a separate file labeled 'Manuscript'.

If you would like to make changes to your financial disclosure, please include your updated statement in your cover letter. Guidelines for resubmitting your figure files are available below the reviewer comments at the end of this letter.

If applicable, we recommend that you deposit your laboratory protocols in protocols.io to enhance the reproducibility of your results. Protocols.io assigns your protocol its own identifier (DOI) so that it can be cited independently in the future. For instructions see: https://journals.plos.org/plosone/s/submission-guidelines#loc-laboratory-protocols. Additionally, PLOS ONE offers an option for publishing peer-reviewed Lab Protocol articles, which describe protocols hosted on protocols.io. Read more information on sharing protocols at https://plos.org/protocols?utm_medium=editorial-email&utm_source=authorletters&utm_campaign=protocols.

As the corresponding author, your ORCID iD is verified in the submission system and will appear in the published article. PLOS supports the use of ORCID, and we encourage all coauthors to register for an ORCID iD and use it as well. Please encourage your coauthors to verify their ORCID iD within the submission system before final acceptance, as unverified ORCID iDs will not appear in the published article. *Only*  the individual author can complete the verification step; PLOS staff *cannot*  verify ORCID iDs on behalf of authors.

We look forward to receiving your revised manuscript.

Kind regards,

Stephan Doering, M.D.

Academic Editor

PLOS One

Journal Requirements:

When submitting your revision, we need you to address these additional requirements.

1. Please ensure that your manuscript meets PLOS ONE's style requirements, including those for file naming. The PLOS ONE style templates can be found at

https://journals.plos.org/plosone/s/file?id=wjVg/PLOSOne_formatting_sample_main_body.pdf and

https://journals.plos.org/plosone/s/file?id=ba62/PLOSOne_formatting_sample_title_authors_affiliations.pdf

2. We note that you have indicated that there are restrictions to data sharing for this study. For studies involving human research participant data or other sensitive data, we encourage authors to share de-identified or anonymized data. However, when data cannot be publicly shared for ethical reasons, we allow authors to make their data sets available upon request. For information on unacceptable data access restrictions, please see http://journals.plos.org/plosone/s/data-availability#loc-unacceptable-data-access-restrictions.

Before we proceed with your manuscript, please address the following prompts:

a) If there are ethical or legal restrictions on sharing a de-identified data set, please explain them in detail (e.g., data contain potentially identifying or sensitive patient information, data are owned by a third-party organization, etc.) and who has imposed them (e.g., a Research Ethics Committee or Institutional Review Board, etc.). Please also provide contact information for a data access committee, ethics committee, or other institutional body to which data requests may be sent.

b) If there are no restrictions, please upload the minimal anonymized data set necessary to replicate your study findings to a stable, public repository and provide us with the relevant URLs, DOIs, or accession numbers. Please see http://www.bmj.com/content/340/bmj.c181.long for guidelines on how to de-identify and prepare clinical data for publication. For a list of recommended repositories, please see https://journals.plos.org/plosone/s/recommended-repositories. You also have the option of uploading the data as Supporting Information files, but we would recommend depositing data directly to a data repository if possible.

Please update your Data Availability statement in the submission form accordingly.

3. In this instance it seems there may be acceptable restrictions in place that prevent the public sharing of your minimal data. However, in line with our goal of ensuring long-term data availability to all interested researchers, PLOS’ Data Policy states that authors cannot be the sole named individuals responsible for ensuring data access (http://journals.plos.org/plosone/s/data-availability#loc-acceptable-data-sharing-methods).

Data requests to a non-author institutional point of contact, such as a data access or ethics committee, helps guarantee long term stability and availability of data. Providing interested researchers with a durable point of contact ensures data will be accessible even if an author changes email addresses, institutions, or becomes unavailable to answer requests.

Before we proceed with your manuscript, please also provide non-author contact information (phone/email/hyperlink) for a data access committee, ethics committee, or other institutional body to which data requests may be sent. If no institutional body is available to respond to requests for your minimal data, please consider if there any institutional representatives who did not collaborate in the study, and are not listed as authors on the manuscript, who would be able to hold the data and respond to external requests for data access? If so, please provide their contact information (i.e., email address). Please also provide details on how you will ensure persistent or long-term data storage and availability.

4. If the reviewer comments include a recommendation to cite specific previously published works, please review and evaluate these publications to determine whether they are relevant and should be cited. There is no requirement to cite these works unless the editor has indicated otherwise.

[Note: HTML markup is below. Please do not edit.]

Reviewers' comments:

Reviewer's Responses to Questions

**Comments to the Author**

1. Is the manuscript technically sound, and do the data support the conclusions?

Reviewer #1: Yes

Reviewer #2: Yes

**********

2. Has the statistical analysis been performed appropriately and rigorously?

Reviewer #1: Yes

Reviewer #2: Yes

**********

3. Have the authors made all data underlying the findings in their manuscript fully available?

The PLOS Data policy

Reviewer #1: No

Reviewer #2: Yes

**********

4. Is the manuscript presented in an intelligible fashion and written in standard English?

Reviewer #1: Yes

Reviewer #2: Yes

**********

Reviewer #1: While I do have some knowledge about adolescent mental health, the topic and context of this particular paper is outside my area of expertise. I am also not an expert in qualitative methods or research. I do hope that the editors have invited reviewers who know the filed better than me.

Having said that, I find the paper well-written, thorough, and mostly clear. My comments (in no particular order):

The perhaps most major comment concerns the mixed methods approach. While this is commendable, and provides opportunities for synergy effects, I think the approach is underdeveloped in this case. The two analyses mostly appear as parallel analyses, with very little cross-breeding. This is especially evident in the discussion-section, where the results of the qualitative analysis are only mentioned in two sentences (lines 373-375). Overall, the qualitative analysis is relatively more neglected and appears to be somewhat of a sideshow. I suggest greater integration between the quantitative and qualitative parts, or alternatively, dropping the qualitative one.

Only adolescents who scored high on specific questionnaires (PHQ-D > 10, GAD-7 > 5, 156 SSS-8 > 8) were invited to participate in the interviews. What is the motivation for this selection strategy? What do the authors gain (or miss) from focusing on this group?

The description of the qualitative method is inadequate and insufficient. The method (Kuckhartz, which I don’t know anything about) is described in one sentence (lines 172-174). I can understand that specific qualitative methods are more difficult to describe in a general way (they are less standardized), but readers need more context here.

The connection between the four categories and the aim of “identifying psychosocial situations and contexts in which the adolescent experiences psychosocial stress” (lines 143-144) is not clear to me. This is particularly evident in relation to the “somatic complaints”- and “informational aspects”-categories: how do these categories address the aim? Likewise, the “psychological aspects”- and the ”structural problems”-categories seems to be about causes of psychosocial stress, not contexts. Moreover, the “somatic complaints”-category seems underdeveloped to me.

The authors make a big deal about the “transition-framing” (e.g., “during the transition period”, “transition process”, “throughout the transition phase”). These terms, including transition itself, imply a temporal process: a move, in time, from one state to another. It is not clear to me in what way the design of study, given the cross-sectional data, really addresses this issue. It appears more as a snapshort of the situation at the beginning of a transition period. While this is also a valid topic, right now there is a discordance between the design and the framing of the study.

Minor comments:

Tables 2 and 3 may be integrated into one for improved clarity and efficiency.

There appears to be a few minor errors in the writing that the authors (or an LLM) can probably pick up with a close reading of the manuscript.

Reviewer #2: Dear author, first of all, I am very pleased to review your valuable work.

*The objective and study design are not clearly stated in the abstract.

*The methodology section might be more structured if presented under headings like those below.

Study design

Research questions or Hypothesis

Data collection tools

Collection of Data

Statistical analysis

Ethical Aspect of Research

Study limitation

Strenght of the study

*In the measurement section, no information is provided regarding whether validity and reliability analyses were conducted for the scales or concerning the validity and reliability coefficients; it would be appropriate to include this information, citing the relevant references.

*Since multiple scales are used in the study, it is recommended to identify the research questions individually and write the discussion section using headings based on these questions. This would allow for a more systematic discussion.

*Incorporating the results of recent intervention studies addressing anxiety, ineffective role performance, family-centered care, social interaction and lack of information about ilness in adolescents with chronic illnesses could enrich the discussion. You may utilize the results section of the recent example study provided below.

Güneş EO and Cetinkaya S (2026) The use of the Roy Adaptation Model in clinical nursing care of an adolescent diagnosed with type 1 diabetes (case report). Front. Clin. Diabetes Healthc. 7:1721420. doi: 10.3389/fcdhc.2026.1721420.

**********

what does this mean?). If published, this will include your full peer review and any attached files.

If you choose “no”, your identity will remain anonymous but your review may still be made public.

**Do you want your identity to be public for this peer review?**  For information about this choice, including consent withdrawal, please see our Privacy Policy

Reviewer #1: No

Reviewer #2: **Yes:**  Ecem Oksal Güneş

**********

[NOTE: If reviewer comments were submitted as an attachment file, they will be attached to this email and accessible via the submission site. Please log into your account, locate the manuscript record, and check for the action link "View Attachments". If this link does not appear, there are no attachment files.]

To ensure your figures meet our technical requirements, please review our figure guidelines: https://journals.plos.org/plosone/s/figures

You may also use PLOS’s free figure tool, NAAS, to help you prepare publication quality figures: https://journals.plos.org/plosone/s/figures#loc-tools-for-figure-preparation.

NAAS will assess whether your figures meet our technical requirements by comparing each figure against our figure specifications.

# Author comment: Author response to Decision Letter 1

**DOI:** 10.1371/journal.pone.0359441.r002

25 Aug 2026

Revision of the Manuscript PONE-D-25-54330: Psychosomatic Symptom Burden and Family Functioning of Adolescents with Chronic Diseases in Transition to Adult Medicine – Implications for Psychosocial Support

Dear Prof. Dr. Doering, dear Editorial Board,

Enclosed please find the revision of the above manuscript.

We appreciate the reviewer’s careful evaluation of the manuscript and have addressed the comments in the revised manuscript. The changes made in the manuscript are highlighted. The data generated or analysed during this study are included in this published article and its supplementary information file (supplemental Table S4).

Please see below our detailed response to the reviewer’s concerns.

Response to the comments of reviewer 1:

While I do have some knowledge about adolescent mental health, the topic and context of this particular paper is outside my area of expertise. I am also not an expert in qualitative methods or research. I do hope that the editors have invited reviewers who know the filed better than me.

Having said that, I find the paper well-written, thorough, and mostly clear. My comments (in no particular order):

1. The perhaps most major comment concerns the mixed methods approach. While this is commendable, and provides opportunities for synergy effects, I think the approach is underdeveloped in this case. The two analyses mostly appear as parallel analyses, with very little cross-breeding. This is especially evident in the discussion-section, where the results of the qualitative analysis are only mentioned in two sentences (lines 373-375). Overall, the qualitative analysis is relatively more neglected and appears to be somewhat of a sideshow. I suggest greater integration between the quantitative and qualitative parts, or alternatively, dropping the qualitative one.

We agree with the reviewer that the qualitative results were not adequately integrated into the discussion. In the revised manuscript we have inserted a paragraph to discuss the qualitative results and relate them to the results of the quantitative analysis (please see lines 415-428).

2. Only adolescents who scored high on specific questionnaires (PHQ-D > 10, GAD-7 >5, SSS-8 > 8) were invited to participate in the interviews. What is the motivation for this selection strategy? What do the authors gain (or miss) from focusing on this group?

We selected adolescents or young adults with a high score in depression, anxiety, or somatic symptom severity because we hypothesized that these persons would be most likely to talk about problem areas and describe their support needs. However, we agree with the reviewer that with this selection strategy we also missed out on the thoughts of less burdened chronically ill adolescents and young adults. We have now included this as a limitation of the interview study (lines 448-451).

3. The description of the qualitative method is inadequate and insufficient. The method (Kuckhartz, which I don’t know anything about) is described in one sentence (lines 172-174). I can understand that specific qualitative methods are more difficult to describe in a general way (they are less standardized), but readers need more context here.

We have now described the qualitative content analysis in more detail (please see methods section, lines 181-209).

4. The connection between the four categories and the aim of “identifying psychosocial situations and contexts in which the adolescent experiences psychosocial stress” (lines 143-144) is not clear to me. This is particularly evident in relation to the “somatic complaints”- and “informational aspects”-categories: how do these categories address the aim? Likewise, the “psychological aspects”- and the ”structural problems”-categories seems to be about causes of psychosocial stress, not contexts. Moreover, the “somatic complaints”-category seems underdeveloped to me.

We have now clarified the aim of the interview (lines 156-158). We also clarified the main research question that determined the qualitative content analysis (lines 192-193).

5. The authors make a big deal about the “transition-framing” (e.g., “during the transition period”, “transition process”, “throughout the transition phase”). These terms, including transition itself, imply a temporal process: a move, in time, from one state to another. It is not clear to me in what way the design of study, given the cross-sectional data, really addresses this issue. It appears more as a snapshort of the situation at the beginning of a transition period. While this is also a valid topic, right now there is a discordance between the design and the framing of the study.

It is correct that the study was cross-sectional, capturing one time point in the process of transition. We have now clarified this in the revised manuscript. We have also shortened the description of the transition process to underline that we focus on the beginning of the transition.

Minor comments:

6. Tables 2 and 3 may be integrated into one for improved clarity and efficiency.

We have integrated tables 2 and 3 into one table.

7. There appears to be a few minor errors in the writing that the authors (or an LLM) can probably pick up with a close reading of the manuscript.

We have now re-checked the manuscript for language errors .

Response to the comments of the reviewer 2:

Dear author, first of all, I am very pleased to review your valuable work.

Major comments:

1. The objective and study design are not clearly stated in the abstract.

We have inserted in the abstract the objective of the study (line 29-30): “The aim was to evaluate psychosocial burden, family functioning, knowledge of their illnesses and identify needs for support”

The methodology section might be more structured if presented under headings like those below.

Study design

Research questions or Hypothesis

Data collection tools

Collection of Data

Statistical analysis

We have restructured the methodology section according to the reviewer’s suggestion.

2. In the measurement section, no information is provided regarding whether validity and reliability analyses were conducted for the scales or concerning the validity and reliability coefficients; it would be appropriate to include this information, citing the relevant references.

We have now included information regarding reliability coefficients and validity of the questionnaires (lines 113-116; 121; 126-127; 132-133; 139-141).

3. Since multiple scales are used in the study, it is recommended to identify the research questions individually and write the discussion section using headings based on these questions. This would allow for a more systematic discussion.

We have now divided the discussion into sections and added different subheadings.

4. Incorporating the results of recent intervention studies addressing anxiety, ineffective role performance, family-centered care, social interaction and lack of information about ilness in adolescents with chronic illnesses could enrich the discussion. You may utilize the results section of the recent example study provided below.

We have inserted in the discussion a paragraph which addresses recent intervention studies (line 434-440): “An appropriate intervention of adaptation for adolescents with chronic diseases could be the Roy Adaptation Model in clinical nursing care to improve adaptation responses in psychological, self-concept, role function, and independence modes (34). For example, in a young adolescent diagnosed with type 1 diabetes, it was demonstrated that the use of the Roy Adaptation Model was appropriate and beneficial, considering that the most common problems among adolescents hospitalized for T1D are physical, psychological, and psychosocial adaptation to the illness (35).”

After all comments have been addressed, we will be very happy if our manuscript can be considered for publication in PLOS One.

On behalf of all the authors,

Yours sincerely,

Daniela Choukair

Consultant Pediatric Endocrinology and Diabetology

Medical Coordinator of the Center for Rare Diseases

Attachment Submitted filename: Reponse to Reviewers.doc

# Editor report: Decision Letter 1

**DOI:** 10.1371/journal.pone.0359441.r003

**Authors:** Stephan Doering

14 Sep 2026

Psychosomatic Symptom Burden and Family Functioning of Adolescents with Chronic Diseases in Transition to Adult Medicine – Implications for Psychosocial Support

PONE-D-25-54330R1

Dear Dr. Choukair,

We’re pleased to inform you that your manuscript has been judged scientifically suitable for publication and will be formally accepted for publication once it meets all outstanding technical requirements.

Within one week, you’ll receive an e-mail detailing the required amendments. When these have been addressed, you’ll receive a formal acceptance letter and your manuscript will be scheduled for publication.

An invoice will be generated when your article is formally accepted. Please note, if your institution has a publishing partnership with PLOS and your article meets the relevant criteria, all or part of your publication costs will be covered. Please make sure your user information is up-to-date by logging into Editorial Manager at Editorial Manager® and clicking the ‘Update My Information' link at the top of the page. For questions related to billing, please contact billing support.

If your institution or institutions have a press office, please notify them about your upcoming paper to help maximize its impact. If they’ll be preparing press materials, please inform our press team as soon as possible -- no later than 48 hours after receiving the formal acceptance. Your manuscript will remain under strict press embargo until 2 pm Eastern Time on the date of publication. For more information, please contact onepress@plos.org.

Kind regards,

Stephan Doering, M.D.

Academic Editor

PLOS One

Additional Editor Comments (optional):

Reviewers' comments:

# Editor report: Acceptance letter

**DOI:** 10.1371/journal.pone.0359441.r004

**Authors:** Stephan Doering

PONE-D-25-54330R1

PLOS One

Dear Dr. Choukair,

I'm pleased to inform you that your manuscript has been deemed suitable for publication in PLOS One. Congratulations! Your manuscript is now being handed over to our production team.

At this stage, our production department will prepare your paper for publication. This includes ensuring the following:

* All references, tables, and figures are properly cited

* All relevant supporting information is included in the manuscript submission,

* There are no issues that prevent the paper from being properly typeset

You will receive further instructions from the production team, including instructions on how to review your proof when it is ready. Please keep in mind that we are working through a large volume of accepted articles, so please give us a few days to review your paper and let you know the next and final steps.

Lastly, if your institution or institutions have a press office, please let them know about your upcoming paper now to help maximize its impact. If they'll be preparing press materials, please inform our press team within the next 48 hours. Your manuscript will remain under strict press embargo until 2 pm Eastern Time on the date of publication. For more information, please contact onepress@plos.org.

You will receive an invoice from PLOS for your publication fee after your manuscript has reached the completed accept phase. If you receive an email requesting payment before acceptance or for any other service, this may be a phishing scheme. Learn how to identify phishing emails and protect your accounts at https://explore.plos.org/phishing.

If we can help with anything else, please email us at customercare@plos.org.

Thank you for submitting your work to PLOS One and supporting open access.

Kind regards,

PLOS One Editorial Office Staff

on behalf of

Professor Stephan Doering

Academic Editor

PLOS One
